# New diagnostic reporting format for endometrial cytology based on cytoarchitectural criteria

**DOI:** 10.1111/j.1365-2303.2008.00581.x

**Published:** 2009-12

**Authors:** K Yanoh, Y Norimatsu, Y Hirai, N Takeshima, A Kamimori, Y Nakamura, K Shimizu, T K Kobayashi, T Murata, T Shiraishi

**Affiliations:** * Departments of Gynaecology, Suzuka General HospitalMie; § Departments of Pathology & Laboratory Medicine, Suzuka General HospitalMie; † Department of Pathology, Kurashiki Central HospitalOkayama; ‡ Department of Gynaecologic Oncology, Cancer Institute Hospital AriakeTokyo, Japan; ¶ Department of Pathology, Saiseikai Noe Hospital, Imperial Gift Foundation Inc.Osaka; ** Department of Pathology, Saiseikai Shiga Hospital, Imperial Gift Foundation Inc.Shiga; †† Department of Pathologic Oncology, School of Medicine, Mie UniversityMie, Japan

**Keywords:** cytodiagnosis, endometrial cytology, reporting format, specimen adequacy, diagnostic criteria, architectural features

## Abstract

**Objective:**

The aim of this study was to develop a new reporting format for endometrial cytology that would standardize the diagnostic criteria and the terminology used for reporting.

**Methods:**

In previous studies, cytoarchitectural criteria were found to be useful for the cytological assessment of endometrial lesions. To apply these criteria, an appropriate cytological specimen is imperative. In this article, the requirements of an adequate endometrial cytological specimen for the new diagnostic criteria are first discussed. Then, the diagnostic criteria, standardized on a combination of conventional and cytoarchitectural criteria, are presented. Third, terminology that could be used, not only for reporting the histopathological diagnosis, but also for providing better guidance for the gynaecologist to determine further clinical action, is introduced. The proposed reporting format was investigated using endometrial cytology of 58 cases that were cytologically underestimated or overestimated compared to the histopathological diagnosis made on the subsequent endometrial biopsy or surgical specimens.

**Results:**

Of the 58 cases, 12 were reassessed as being unsatisfactory for evaluation. Among the remaining 46 cases, 25 of the 27 cases, which had been underestimated and subsequently diagnosed as having endometrial carcinoma or a precursor stage on histopathological examination,were reassessed as recommended for endometrial biopsy. On the other hand, 19 cases overestimated by cytology were all reassessed as not requiring biopsy.

**Conclusions:**

The reporting format for endometrial cytology proposed in this article may improve diagnostic accuracy and reduce the number of patients managed inappropriately.

## Introduction

Compared to cervical cytology, endometrial cytology is considered unreliable, diagnostically. The main reason for this is thought to be the inconspicuous differences between normal and abnormal endometrial cells because of the small size of the cells. The changes in the cellular features and the presence of cell clumps during the normal menstrual cycle complicate matters further. Recently, in an attempt to address these problems, there has been increasing interest in the cytoarchitectural endometrial criteria. However, the practical application of these criteria has not been fully worked out. Usually, endometrial cytology reports are classified into three groups: negative, suspicious or positive. A presumptive histopathological diagnosis may also be reported. However, using this reporting method, many of the suspicious cases are false positives, leading to unnecessary biopsies. In Japan, when atypical endometrial hyperplasia or carcinoma is suspected on cytology, biopsies are usually performed. On the other hand, when normal or atrophic endometrium is reported, no additional biopsies are required, unless carcinoma or a precursor lesion cannot be excluded on clinical grounds. Therefore, for the cytological diagnosis of the endometrium, complete agreement between the cytology and the histopathology is not essential. What matters is whether a biopsy is necessary to establish the final diagnosis.

Currently, for endometrial lesions, there are several quantitative and qualitative cytological methods that are based on the cell collecting techniques of individual laboratories or hospitals.^1^–^5^ However, there is no inclusive diagnostic and reporting system for endometrial cytology that has clear definitions of specimen quality. The purpose of this study was to evaluate an appropriate reporting format that includes a definition of specimen and clinical information adequacy, acceptable diagnostic criteria, and an endometrial cytology report.

## Materials and methods

At the Suzuka General Hospital, the Yamada Red Cross Hospital, and the Yamamoto General Hospital, 12 729 endometrial cytology samples were received between January 2002 and April 2006. Of these cases, 58 were included in this study (Table 1). All 58 specimens had been underestimated or overestimated compared to the subsequent histopathological diagnosis based on endometrial biopsy or surgical specimens, 20 cases having been overestimated cytologically and 38 cases underestimated. In this study, the endometrial cytology obtained by direct sampling of the endometrial cavity were prepared with Endocyte (Laboratoire Ccd, Paris, France), Honestbrush N (Honest Medical, Tokyo, Japan), or Endotube-M (Matsudaikakougyo, Tokyo, Japan).

**Table 1:** Cases undergoing cytological re-assessment

Histological diagnosis	Underestimated	Overestimated
Complex hyperplasia	4	
Atypical simple hyperplasia	3	
Atypical complex hyperplasia	6	
Endometrioid adenocarcinoma	25	
Benign endometrium		
Proliferative endometrium		9
Secretory endometrium		4
Other		6
Endometrial polyp		1
Total	38	20

When Endocyte or Honestbrush N was used, the cell sampler was rolled on a glass slide and the material was spread and smeared. In some cases, to transfer the cells onto the glass slide, the tip of the brush was strongly flicked with the forceps; this was repeated several times as the brush was repositioned along the length of the slide. When the tube was used, the material that had been sucked into the tube was pumped onto the glass slide. All cytological smears were routinely fixed in 95% alcohol and subsequently stained using the standard Papanicolaou method. Tissue samples were routinely formalin fixed, paraffin embedded, and processed for staining with haematoxylin and eosin. The histopathological diagnoses included 20 cases of benign endometrium, four cases of complex hyperplasia, three cases of atypical simple hyperplasia, six cases of atypical complex hyperplasia, and 25 cases of endometrioid adenocarcinoma. In each hospital, these slides were used to evaluate endometrial lesions based on conventional criteria. To assess our reporting system, all cytological and histopathological slides accompanied by the relevant clinical information were re-evaluated microscopically by four of the authors (T.M., K.Y., Y.N., and A.K.). Specimen adequacy was analysed first. Once a specimen was determined to be either satisfactory for evaluation or less than optimal, it was reviewed for cytological evaluation. The following results were obtained with respect to the specimen adequacy criteria and the cytological diagnosis.

### Statement on specimen adequacy

Using the new reporting format, when the maximum diameter of a cell cluster was 0.2 mm or more, it was defined as a cell clump. Specimens that were not acceptable for diagnostic evaluation because of poor fixation, preservation, or labelling, drying of the specimen, an obscure background, distortion of cells or cell clumps, insufficient clinical information or scant cellularity were reported as ‘Unsatisfactory for evaluation’ (Table 2). If the findings were such that the endometrium could be properly assessed despite being an unsatisfactory specimen, for example, when only a few isolated atypical cells were present in a necrotic background or if there was a small clump of obvious carcinoma cells, these specimens were reported as ‘Less than optimal’. In these latter cases, the referring clinician has to recognize that the reported diagnosis may not be reliable. Repeated cytology, endometrial biopsy or clinical follow-up may be chosen depending on the clinical information. All other specimens that had sufficient quality and quantity of material and for which adequate clinical information was available were reported as ‘Satisfactory for evaluation’.

**Table 2:** Unsatisfactory for evaluation

Poor fixation, preservation, or labelling. Dry specimen
Obscuring by inflammation, blood
Distortion of cells or cell clumps at the time of cellpreparation
Lack of or insufficient clinical information age, last menstrual period (age at menopause), genital bleeding, drug use, use of intrauterine device, echo-sonographic findings
Scant cellularity Total cell clumps <10 per one specimen (Cell clump: diameter of cell clump >0.2 mm)

### Diagnostic criteria

Diagnostic criteria consisted of two main elements (Table 3): the criteria that reflect the cytoarchitecture and the conventional criteria (background, atypia of cells, or cell clumps). Cell clumps were classified into two categories: normal cell clumps and abnormal cell clumps. The normal category included cell clumps with a tube or sheet-shaped pattern. The abnormal category included cell clumps with dilated or branched patterns, branched patterns, papillotubular patterns and irregular protrusions. In a previous paper of by Norimatsu *et al.*,^6^ all of these cell clump characteristics were described. Cell clumps composed of metaplastic cells and some irregular small projection figures, usually accompanied by condensed stromal cell clusters,^7^,^8^ were excluded from these four categories as their diagnostic importance is not yet clear.

**Table 3:** Diagnostic criteria for endometrial cytology

(I) Frequency of abnormal cell clump*
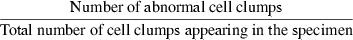
*Endometrial hyperplasia is suspected*: frequency of each abnormal cell clump in total ≥10 and abnormal cell clump ratio ≥10%
*Atypical endometrial hyperplasia or carcinoma is suspected*: frequency of each abnormal cell clump in total ≥10 and abnormal cell clump ratio ≥50%
(II) Background and cellular atypia
Small cell cluster† consisting of atypical cells
Isolated atypical cells
Necrotic background
Metaplastic change [squamous(morule), eosinophilic, ciliated, mucinous, clear cell]

* Exclusive of cell clumps consisting of metaplastic cells.

† maximum diameter of the cell cluster <0.2 mm.

Endometrial hyperplasia was suspected in specimens with a total of 10 or more cell clumps and an abnormal cell clump rate of 20–70%, provided there was no overlap with the over 70% group. Atypical endometrial hyperplasia or endometrial carcinoma was suspected in specimens with a total of 10 or more cell clumps and an abnormal cell clump rate of more than 70%.^9^

The final cytological diagnosis was based on a combination of these results and the conventional criteria.

### The reporting of the endometrial cytological diagnosis

Once the specimen adequacy had been described, the result was categorized into four groups; negative, atypical endometrial cells of undetermined significance (AEC-US), atypical endometrial cells encompassing the spectrum of precursors to endometrial malignant tumour (AEC-PEMT) and Positive. When normal endometrium with proliferative, secretory or, menstrual phase changes or atrophy had been identified cytologically, these cases were reported as ‘negative’. The term AEC-US was used for cases in which benign endometrial disease, such as endometrial bleeding because of ovarian dysfunction, iatrogenic changes, or infection, was suspected. In such cases, subsequent endometrial biopsy is usually not recommended unless the change persists on repeat cytology. The term AEC-PEMT was used for cases in which a pre-malignant lesion, such as atypical endometrial hyperplasia, was suspected and subsequent biopsy was recommended, as for ‘positive’ cases. All options could include additional information suggesting the histopathological diagnosis (Table 4).

**Table 4:** A reporting system for endometrial cytology

Specimen adequacy
Satisfactory for evaluation
Less than optimal
Unsatisfactory for evaluation
Interpretation/result
Negative Proliferative or secretory phase, atrophic endometrium
AEC-US (atypical endometrial cells of undetermined significance) Suspicious for benign endometrial disease (bleeding due to ovarian dysfunction, iatrogenic changes, Infection), or simple endometrial hyperplasia (*biopsy not recommended*)
AEC-PEMT (atypical endometrial cells encompassing the spectrum of precursors to endometrial malignant tumour) Suspicious for complex endometrial hyperplasia, simple or complex atypical endometrial hyperplasia, adenocarcinoma in situ (*biopsy recommended*)
Positive Suspicious for malignant tumour

## Results

The results are summarized in Tables 5–7. With the new reporting format for endometrial cytology, of the 58 cases, 12 cases including eight adenocarcinoma cases were assessed as unsatisfactory for evaluation, 10 cases were assessed as less than optimal and 36 cases were assessed as satisfactory for evaluation. In the 12 cases that were assessed as ‘unsatisfactory for evaluation’, 11 cases were previously underestimated and one case was overestimated. All the 10 cases assessed as ‘less than optimal’ were underestimated. Of 36 cases that were assessed as ‘satisfactory for evaluation’, 17 cases were underestimated and 19 cases were overestimated (Table 5). For the 12 cases found to be unsatisfactory for evaluation, additional sampling was recommended. The most important factor resulting in an unsatisfactory specimen was ‘scant cellularity’ (Table 6). Of the 27 cases originally reported as negative cytologically, 25 were assessed as ‘positive’ or in the AEC-PEMT category. In these cases, biopsy was recommended. The remaining two were assessed as ‘negative’. One of these two cases had atypical simple hyperplasia and the other had atypical complex hyperplasia. Among the cases that had previously been overestimated on the basis of endometrial cytology, none was assessed as ‘recommended endometrial biopsy’ using the new system. Overall, 21 cases were assessed as ‘biopsy not recommended’; all these cases had subsequent biopsies that revealed normal endometrium (Table 7).

**Table 7:** Results of the new reporting system for endometrial cytology (satisfactory for assessment and less than optimal specimens)

	Underestimated	Overestimated	Total
Repeat sampling warranted	0	0	0
Positive or AEC-PEMT(*biopsy recommended*)	25	0	25
Negative or AEC-US (*biopsynot recommended*)	*2	19	21
Total	27	19	46

* Atypical simple hyperplasia: 1 case; Atypical complex hyperplasia: 1 case.

**Table 6:** Unsatisfactory for evaluation

	Underestimated	Overestimated	Total
Obscuring inflammation	1	0	1
Lack of or insufficientclinical information	0	0	0
Scant cellularity	5	0	5
Obscuring by blood orscant cellularity	5	1	6
Total	11	1	12

**Table 5:** Assessment of specimen adequacy using the new reporting system for endometrial cytology

	Underestimated	Overestimated	Total
Unsatisfactory	11	1	12
Less than optimal	10	0	10
Satisfactory	17	19	36
Total	38	20	58

## Discussion

In Japan, after a health insurance law for the elderly was passed in 1987, endometrial cytology became a routine method for the initial examination of endometrial lesions; since then, the importance of endometrial cytology has gradually become recognized. It is rare that a sample is not collected because of sampling difficulty or because of unacceptable pain for the patient. Although endometrial cytology is now commonly used, it has a low diagnostic accuracy. In recent years, it has been reported that, for the cytological assessment of endometrial lesions, the cytoarchitectural characteristics are more useful than conventional criteria that reflect only cellular atypia. In previous studies, the correlation between cytoarchitecture and histopathological structure has been emphasized. Norimatsu *et al.*^6^ found statistically significant differences in the frequency of cell clumps classified according to their cytoarchitectural characteristics among cases with normal proliferative endometrium, endometrial hyperplasia, atypical endometrial hyperplasia and grade 1 endometrioid adenocarcinoma. Moreover, Shimizu *et al.*^7^ and Norimatsu *et al.*^8^ reported that endometrium with dysfunctional uterine bleeding caused by an anovulatory cycle, which contains endometrial glandular and stromal breakdown (EGBD), can be distinguished from endometrial hyperplasia when care is taken to determine the frequency of cell clumps classified according to their cytoarchitectural characteristics and metaplastic changes. Given these facts, a precise assessment of the cytoarchitecture seems to be important. In addition to the quality assessment, a quantitative assessment based on estimating the frequency of each cell type’s cell clumps in a specimen is important.

When using cytoarchitectural criteria and assessing cell quantity for endometrial cytology, standardized specimens and adequate clinical information are necessary. In our new reporting format, first of all, specimen adequacy was assessed as in the Bethesda System to serve as a guideline. In this study, the presence of scant cellularity, obscuring inflammation and blood or both were the most important factors that resulted in unsatisfactory specimens. The sampling and preparation of the specimens were performed by gynaecologists who had to use the cell collection instruments properly and transfer the material collected onto glass slides. Recently, Fujihara *et al.*^10^ advocated that the ‘flick’ method using the Uterobrush was suitable for observing cytoarchitecture and was helpful to standardize the criteria for direct intrauterine cell samples. We also use the brush type instrument with the ‘flick’ method, as it allows a sufficient quantity and quality of the specimen to be placed on glass slides. Sampling and preparing devices must be made for each cell sampler. In the not too distant future, the method of direct intrauterine cell sampling is likely to be standardized so as to allow common diagnostic criteria to be used. Furthermore, liquid-based endometrial cytology may make it possible for a sufficient quantity and quality of a sample to be transferred onto the glass slide more easily. More recently, Norimatsu *et al.*^11^ and Buccoliero *et al.*^12^ reported the successful sampling of endometrial cells using liquid-based preparation as an efficient diagnostic method for excellent cellular preservation. This method is expected to be easier in observation of cell and cell cluster than conventional endometrial cytology. Furthermore, the technique is useful in providing material for adjunctive cytological diagnostic and research purposes. In the foreseeable future, liquid-based endometrial cytology must be adopted.

With this new reporting format for diagnosis in endometrial cytology, we combined the use of the conventional criteria with new criteria, such as the frequency rate of cell clumps, based on cytoarchitectural features. When the total number of abnormal cell clumps observed in a specimen is 10 or more and the abnormal cell clump rate is more than 20%, this suggests endometrial hyperplasia. When the abnormal cell clump rate is more than 70%, this suggests atypical endometrial hyperplasia or endometrial carcinoma. All these diagnostic baseline values have been chosen empirically and hence are open to discussion and may be refined by other investigators on the basis of additional studies. Opinions vary as regards which abnormal cell clump rate should be used for cytomorphology. Moreover, conventional criteria, such as tumour background, cellular or nuclear atypia, small atypical cell clusters and isolated atypical cells, are still useful for diagnosing endometrial cytology, as there are many cases that can be estimated correctly without cytoarchitectural criteria. Nevertheless, diagnostic accuracy can be improved by applying cytoarchitectural criteria together with conventional criteria. The importance of cytoarchitectural criteria cannot be overemphasized. In practice, the diagnosis of endometrial cytology must include these criteria.

In the literature, other diagnostic devices requiring their own cell sampling methods have been described.^1^–^5^,^13^ However, because of the specificity and complexity of their sampling and assessment procedures, they have not been adopted. Moreover, their accuracy for detecting endometrial carcinoma and its precursor lesion is not satisfactory. In contrast, our diagnostic criteria have been independently assessed by other Japanese institutions,^14^,^15^ which reported increased diagnostic accuracy with their use. With all of the laboratories in this study, we achieved an extremely high diagnostic accuracy, as the cytological report focused on the necessity or otherwise of doing an endometrial biopsy. The high accuracy of the reassessed specimens might have been affected by the fact that this study was restricted to cases in which the cytology was overestimated or underestimated. A prospective study using this new reporting format would clarify its validity. Nevertheless, the new format provides uniform diagnostic terminology to improve interlaboratory communications between the laboratory and the clinician. Given our results, it appears that use of the criteria proposed in this paper is a valid and reproducible method for the cytological evaluation of endometrial lesions that can avert unnecessary endometrial biopsies.

In the results, AEC-US and AEC-PEMT were adopted. The use of this category makes it possible to avoid confusion about the meaning of the report. When the cytology was consistent with benign disease, changes due mostly to ovarian dysfunction, including EGBD or simple endometrial hyperplasia, then the cytological report stated that was AEC-US. For whatever reason, as cytologists, we should avoid the risk of AEC-US becoming a ‘wastebasket’ classification encompassing various types of benign glandular changes as well as changes associated with significant cancer precursor lesions. A diagnosis of AEC-US should be qualified to indicate whether a reactive process or AEC-PEMT is more likely. To assist the clinician in determining patient management, the cytologist should communicate as much as possible about the diagnosis and the differential diagnoses under consideration. Appropriate triage strategy may be considered. A presumptive histopathological diagnosis of the endometrial phase is required to make this assessment. Usually, subsequent endometrial biopsy is not recommended in these ‘negative’ cases. When the cytology is consistent with complex endometrial hyperplasia, simple or complex atypical endometrial hyperplasia or adenocarcinoma in situ, the cytological report should state AEC-PEMT. The histopathological diagnosis predicted by the cytology must be included in the interpretation. In these cases, endometrial biopsy is recommended to make the final diagnosis in a ‘positive’ case. This new reporting format is not concerned with cases of simple endometrial hyperplasia because there is no consensus on their treatment. The diagnostic importance of cellular alterations such as eosinophilic cell change is too complicated to be examined in detail in this paper; further investigation is required to resolve this issue.

## Conclusion

The reporting format that has been proposed is intended for use in medical consultation, just as the Bethesda System is used. This format is useful for the proper cytological assessment of endometrial lesions, which is of great benefit to patients. Implementation of this format requires accurate and appropriate clinical information, an adequate specimen that is obtained in a satisfactory and standardized manner and mutual understanding between the pathologist and the gynaecologist. We predict that the field of gynaecological cytology will be of increasing interest to cytopathologists if this reporting format becomes the primary vehicle for communicating diagnoses to clinicians.
